# The prevalence of microsporidia in China : A systematic review and meta-analysis

**DOI:** 10.1038/s41598-019-39290-3

**Published:** 2019-02-28

**Authors:** Luyao Qiu, Wanyuan Xia, Wendao Li, Jing Ping, Songtao Ding, Handeng Liu

**Affiliations:** 1 Department of Cell Biology and Genetics, Experimental Teaching Center, Chongqing Medical University, Chongqing, 400016 P.R. China; 2 College of Pediatrics, Chongqing Medical University, Chongqing, 401331 P.R. China; 3 Department of Health Statistics, School of Public Health and Management, Chongqing Medical University, Chongqing, 400016 P.R. China

**Keywords:** Fungi, Molecular biology, Fungal infection

## Abstract

Microsporidia are a diverse parasite phylum infecting host from all major taxa in all global biomes. This research was conducted to conclude the prevalence of microsporidia in China. All published articles up to February 16, 2018 were considered, including descriptive, cross-sectional, case-control and epidemiology studies. A total of 1052 articles were separated after literature search. After a strict selection according to our criteria, 82 articles were included in qualitative synthesis and ultimately 52 studies were included in quantitative synthesis. Three species of microsporidia were confirmed to exist in China, including *Enterocytozoon bieneusi* (*E*. *bieneusi*), *Nosema* and *Encephalitozoon cuniculi* (*E*. *cuniculi*). The highest overall estimated prevalence of *E*. *bieneusi* in humans was 8.1%, which was observed in acquired immunodeficiency syndrome patients (AIDS). Moreover, the prevalence of *E*. *bieneusi* in animals including the cattle, dogs, pigs, deer, sheep and goats were analyszed in this study. The overall estimated prevalence of *E*. *bieneusi* acquired by using the random effects model in meta-analysis in cattle, dogs, pigs, sheep and goats and deer was 20.0% (95% confidence intervals: 0.133–0.266, *I*^2^ = 98.031%, *p* < 0.0001), 7.8% (95% *CI*: 0.050–0.106, *I*^2^ = 60.822%, *p* = 0.0537), 45.1% (95% *CI*: 0.227–0.674, *I*^2^ = 98.183%, *p* < 0.0001), 28.1% (95% *CI*: 0.146–0.415, *I*^2^ = 98.716%, *p* < 0.0001) and 19.3% (95% *CI*: 0.084–0.303, *I*^2^ = 96.995%, *p* < 0.0001) respectively. The overall detection rate of *E*. *bieneusi* in water acquired by using the random effects model in meta-analysis was 64.5% (95% CI: 0.433–0.857, *I*^2^ = 98.486%, *p* < 0.0001). Currently, 221 genotypes of *E*. *bieneusi*, 1 genotype of *E*. *cuniculi* and 6 *Nosema* were detected in China. The most prevalent genotype of *E*. *bieneusi* was genotype D, followed by BEB6 and EbpC.

## Introduction

Microsporidia, classified as highly specialized fungi, are unicellular and obligate intracellular opportunistic pathogens which can infect a wide range of vertebrate and invertebrate hosts such as fish, insects, farm animals, and companion pets^1,2^. Up to now, the phylum microsporidia is consisting of more than 170 genera and 1300 species. Among these genera, eight of them have been responsible for human infections, including *Enterocytozoon*, *Pleistophora*, *Encephalitozoon*, *Vittaforma*, *Trachipleistophora*, *Brachiola*, *Nosema* and *Microsporidium*^3–5^. *Enterocytozoon bieneusi* (*E*. *bieneusi*) and the *Encephalitozoon* species (*E*. *cuniculi*, *E*. *intestinalis* and *E*. *hellem*) are the four major species infecting humans. *E*. *bieneusi* which is responsible for more than 90% of cases with microsporidiosis in humans is most commonly diagnosed^2,6^. As a zoonotic pathogen, the main transmission way of *E*. *bieneusi* is fecal-oral route or oral-oral route because its spores are shed into environment via feces. Therefore, the way of consumption of contaminated food and water is the main route of *E*. *bieneusi* infection^7^. In addition, most microsporidial infections have been reported to occur in severely immunocompromised individuals, mainly HIV/AIDS patients, but cases in HIV-negative people, including travelers and elderly people, are continually increasing. These pathogens could cause a variety of systemic and nonsystemic diseases, and the most common clinical manifestation is chronic diarrhea. For immunocompromised patients such as AIDS, organ transplant recipients and cancer, the infection could lead to life-threatening diarrhea and weight loss^4^. For healthy individuals, these pathogens could cause self-limiting diarrhea and malabsorption^4^. Concerning extra-intestinal infections, *Encephalitozoon* spp. was able to disseminate to many other organs and tissues of the body^8^. It was confirmed that *E*. *biensusi* had potential for infections like pneumonia, while *Brachiola algerae*, *Nosema ocularum*, *Trachipleistophora hominis* and *Encephalitozoon* species (*E*. *cuniculi*, *Encephalitozoon hellem*, and *Encephalitozoon intestinalis*) were associated with keratoconjonctivitis^5^. In terms of therapy, albendazole is effective against *E*. *intestinalis* but not on infection with *E*. *bieneusi*. Though Fumagillin has shown the clinical therapeutic effect on *E*. *bieneusi*^5^, its efficacy is counterbalanced by its adverse effects^5^. Over the last decade, PCR amplification and staining techniques have been the most common approaches for detection, when sequence analysis of the internal transcribed spacer (ITS) has been widely used in characterizing the molecular epidemiology^9^. To this end, this study was carried out a systematic review with meta-analysis of microsporidia studies in vertebrate and invertebrate hosts and Chinese population distributed in different regions in China. The present study may be the first meta-analysis that provides overall results based on available molecular and staining methods. According to this systematic review, not only we can improve awareness about microsporidia prevalence in various regions of China, but we will also be able to implement better preventive and treatment strategies.

## Results

**Figure 1:**
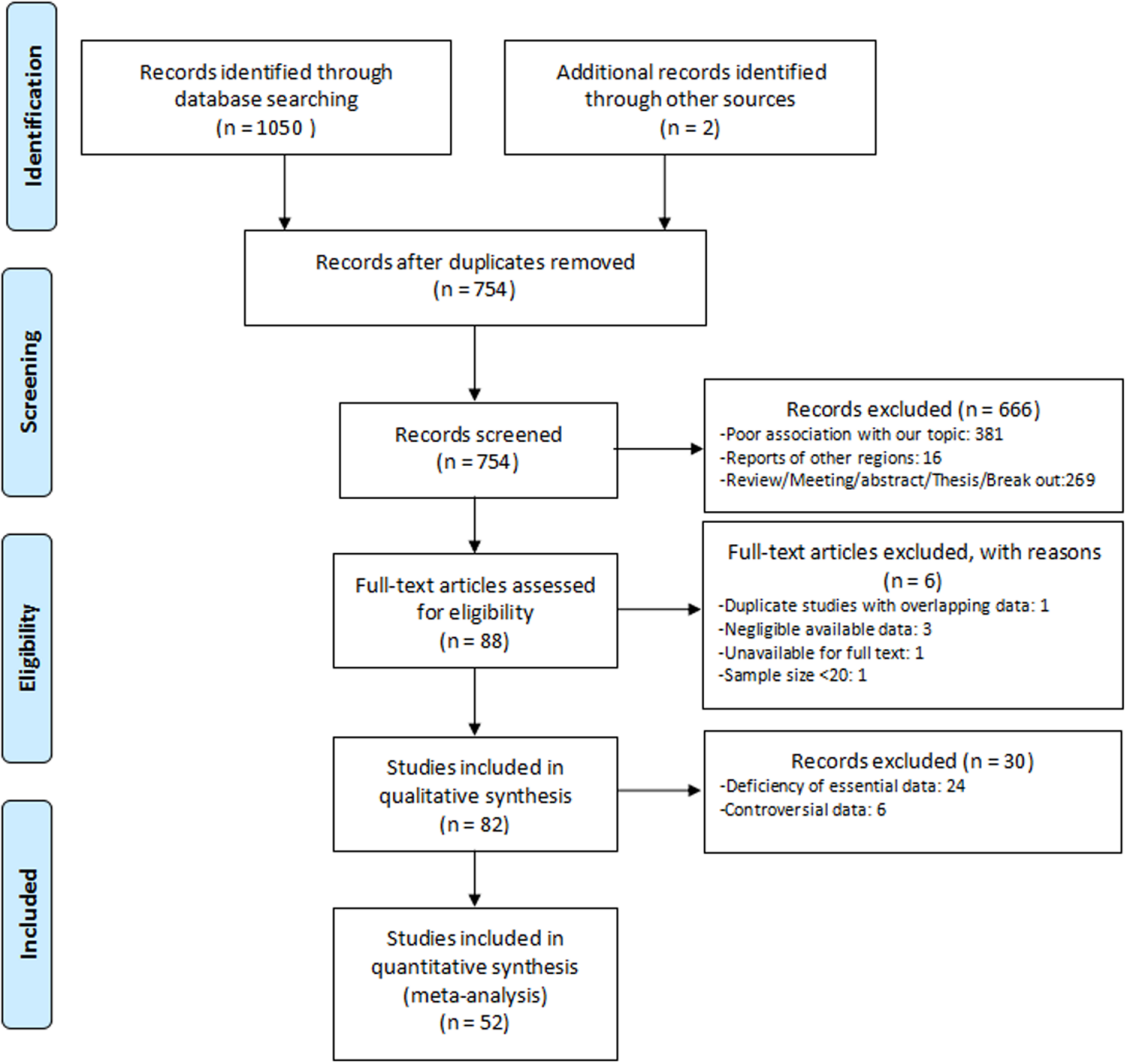
Flowchart of study selection.

A total of 1050 articles were separated after literature search of 8 databases (Pubmed: 19, Embase: 60, Web of Science: 151, Cochrane library: 0, CNKI: 403, Wanfang: 289, VIP: 128, CBM: 0), with 2 articles coming from other sources, and ultimately 82 articles were included in qualitative synthesis (Fig. 1). After excluding 6 studies with controversial data and 24 studies with essential data deficiency, 52 studies were included in quantitative synthesis (meta-analysis). All data extracted from included articles was demonstrated in Table 1 and Supplementary Table S1 to S4. As few articles have reported the prevalence of *Nosema*^10,11^ and *Encephalitoznoon* spp.^12–14^, it is not clear about their real prevalence. Thus, our further analysis only emphasised on *E*. *bieneusi*. Moreover, our statistical analysis of *E*. *bieneusi* was confined to human-beings, water, cattle, dogs, pigs, deer and sheep and goats.

**Table 1:** Prevalence of *E*.*bieneusi* in humans in China (C-S: case-control study; C-C: cross-sectional study).

Author	Year	Region	Positive Cases	Prevalence(%)	Detection method	Genus&Species	Score	Type of syudy	Reference
**Diarrhea individiuals**									
Zhang *et al*.	2011	Jilin	9	22.5	PCR	*E*.*bieneusi*	2	C-S	^15^
Chen *et al*.	2012	Shanghai	2	1.8	Staining	—	3	C-S	^24^
Yang *et al*.	2014	Heilongjiang	1	25.0	PCR	*E*.*bieneusi*	3	C-S	^25^
Liu *et al*.	2014	Shanghai	34	13.5	PCR	*E*.*bieneusi*	4	C-S	^26^
Zhang *et al*.	2017	Heilongjiang	4	3.6	PCR	*E*.*bieneusi*	3	C-S	^27^
Wang *et al*.	2017	Hubei	1	0.2	PCR	*E*.*bieneusi*	4	C-S	^28^
Qiu *et al*.	2017	Sichuan	92	7.6	Staining&PCR	*E*.*bieneusi*	2	C-S	^12^
Qiu *et al*.	2017	Sichuan	92	3.3	Staining&PCR	*E*.*intestinals*	2	C-S	^12^
Qiu *et al*.	2017	Sichuan	92	0	Staining&PCR	*E*.*cuniculi*	2	C-S	^12^
Qiu *et al*.	2017	Chongqing	32	6.3	Staining&PCR	*E*.*bieneusi*	2	C-S	^12^
Qiu *et al*.	2017	Chongqing	32	3.1	Staining&PCR	*E*.*intestinals*	2	C-S	^12^
Qiu *et al*.	2017	Chongqing	32	0	Staining&PCR	*E*.*cuniculi*	2	C-S	^12^
**AIDS patients**									
Wang *et al*.	2013	Henan	39	5.7	PCR	*E*.*bieneusi*	3	C-S	^29^
Xie *et al*.	2015	Hunan	8	5.3	Staining	—	1	C-S	^30^
Liu *et al*.	2017	Guangxi	33	11.6	PCR	*E*.*bieneusi*	3	C-C	^31^
**Other patients**									
Chen *et al*.	2014	Shanghai	44	0.7	Staining	—	4	C-S	^32^
Yang *et al*.	2014	Heilongjiang	8	22	PCR	*E*.*bieneusi*	3	C-S	^25^
Zhang *et al*.	2017	Heilongjiang	1	0.4	PCR	*E*.*bieneusi*	3	C-C	^27^
**Normal immunity individuals**									
Wang *et al*.	2013	Henan	29	4.2	PCR	*E*.*bieneusi*	3	C-S	^29^
Yang *et al*.	2014	Heilongjiang	10	4.7	PCR	*E*.*bieneusi*	3	C-S	^25^
Liu *et al*.	2017	Guangxi	0	0	PCR	*E*.*bieneusi*	3	C-C	^31^

### Prevalence of *E*. *bieneusi* in human-beings

**Figure 2:**
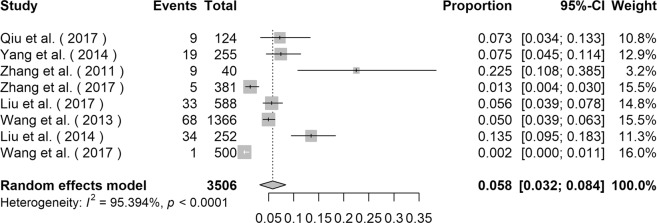
Forest plot diagram showing *E*. *bieneusi* infection in human-beings.

The prevalence of *E*. *bieneusi* in human-beings varied from 0.2% to 22.5% (Table 1), and the highest infection rate was observed in children from Jilin province^15^. The overall prevalence of *E*. *bieneusi* acquired by using the random effects model in meta-analysis was 5.8% (95% *CI*: 0.032–0.084, *I*^2^ = 95.394%, *p* < 0.0001, Fig. 2) among 3506 individuals. Subgroup analysis of gender, sampling region and stool appearance indicated that gender, sampling region and stool appearance were not the source of heterogeneity (Table 2). The estimated overall prevalence of males was 5.0% (95% *CI*: 0.019–0.082, *I*^2^ = 92.403%, *p* < 0.0001, Table 2) and that of females was 4.6% (95% *CI*: 0.011–0.080, *I*^2^ = 86.151%, *p* < 0.0001, Table 2). The estimated overall prevalence of *E*. *bieneusi* in Southern China was 6.4% (95% *CI*: 0.016–0.112, *I*^2^ = 94.733%, *p* < 0.0001, Table 2) and that of Northern China was 5.5% (95% *CI*: 0.020–0.089, *I*^2^ = 91.287%,* p* < 0.0001, Table 2). For diarrheal patients, AIDS patients, other patients and healthy individuals, the overall prevalence of *E*. *bieneusi* was 6.4% (95% *CI*: 0.026–0.150, *I*^2^ = 87.013%, *p* < 0.0001, Table 2), 8.1% (95% *CI*: 0.040–0.159, *I*^2^ = 89.666%, *p* = 0.0019, Table 2), 3.5% (95% *CI*: 0.001–0.716, *I*^2^ = 93.834%, *p* < 0.0001, Table 2) and 3.6% (95% *CI*: 0.018–0.072, *I*^2^ = 63.534%, *p *= 0.0644, Table 2) respectively.

**Table 2:** Subgroup analysis of *E*.*bieneusi* infection in humans (gender, region and stool appearance).

Factor	Total individuals	Positive cases	Overall prevalence (%) (95% CI)	P-values	I-squared	Reference
Gender				<0.0001	89.210%	^25–28,31^
Male	965	55	5.0 (0.019–0.082)	<0.0001	92.403%	
Female	668	28	4.6 (0.011–0.080)	<0.0001	86.151%	
Region				<0.0001	94.737%	^1–11^
Southern China	1464	77	6.4 (0.016–0.112)	<0.0001	94.733%	
Northern China	2042	101	5.5 (0.020–0.089)	<0.0001	91.287%	
Stool appearance				<0.0001	87.643%	^12,15,25–29,31^
Diarrhea	1026	57	6.4 (0.026–0.150)	<0.0001	87.013%	
HIV/AIDS patients	968	72	8.1 (0.040–0.159)	0.0019	89.666%	
Other patients	307	9	3.5 (0.001–0.716)	<0.0001	93.834%	
Normal immunity patients	1201	39	3.6 (0.018–0.072)	0.0664	63.534%	

### Prevalence of *E*. *bieneusi* in cattle

**Figure 3:**
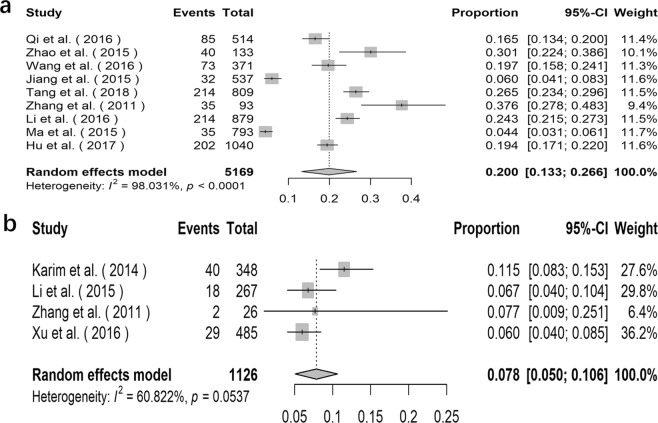
Forest plot diagram showing *E*. *bieneusi* infection in cattle and dogs (**a** cattle; **b** dogs).

The prevalence of *E*. *bieneusi* in cattle varied from 2.0% in Shandong province to 46.8% in Ningxia Hui Autonomous Region (Supplementary Data Table 1). The overall prevalence of *E*. *bieneusi* acquired by using the random effects model in meta-analysis was 20.0% (95% *CI*: 0.133–0.266, *I*^2^ = 98.031%, *p* < 0.0001, Fig. 3a). Totally, 40 genotypes of *E*. *bieneusi* were detected in cattle in China, including NECA1, NECA2, NECA3, NECA4, NECA5, NESH5, O, I, J, D, H, N, EbpA, EbpC, CC4, BEB4, BEB6, BEB8, CD6, CM8, COS-I, CHC1, CHC2, CHC3, CHC4, CHC5, CHC6, CHC7, CHC8, CHG2, CHG3, CHN13, CHN14, CHN11, CHN12, CHN4, CHN15, WCY1, CSX1, and CSX2 (Supplementary Data Table 2).

### Prevalence of *E*. *bieneusi* in dogs

Covering 5 provinces and 2 municipalities, four studies concentrated on the infection of dogs^15–18^. The infection rate varied from 6.0% in Shanghai to 25.0% in Sichuan (Supplementary Data Table 1). The overall prevalence of *E*. *bieneusi* acquired by using the random effects model in meta-analysis was 7.8% (95% *CI*: 0.050–0.106, *I*^2^ = 60.822%, *p* = 0.0537, Fig. 3b). Totally, 24 genotypes of *E*. *bieneusi* were detected in dogs, including PtEbIX, EbpC, D, NED1, NED2, NED3, NED4, CD1, CD2, CD3, CD4, CD5, CD6, CD7, CD8, CD9, CM1, Peru8, EbpA, O, PigEBITS5, type IV, CHN5 and CHN6 (Supplementary Data Table 2).

### Prevalence of *E*. *bieneusi* in pigs

**Figure 4:**
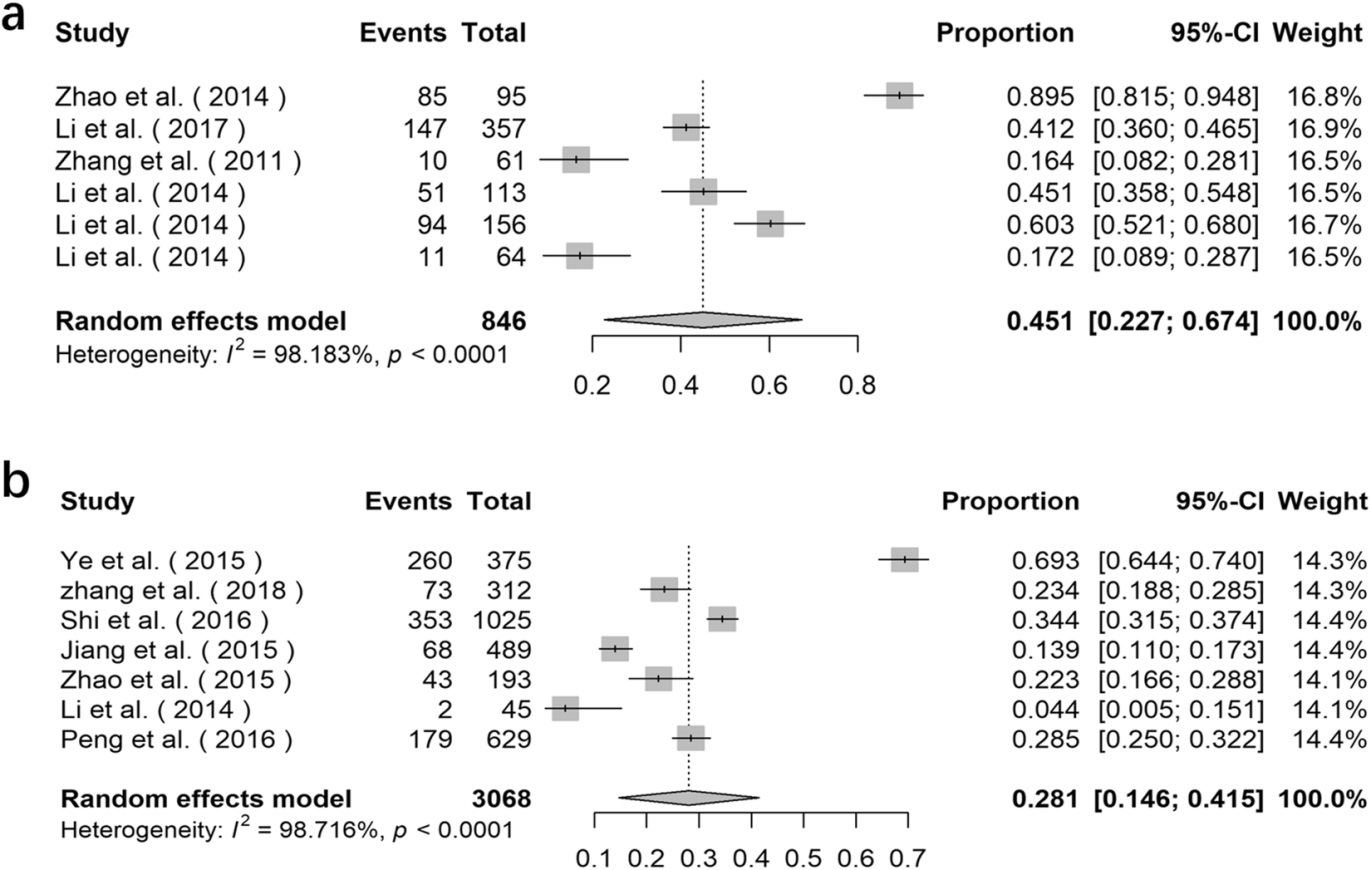
Forest plot diagram showing *E*. *bieneusi* infection in pigs and sheep and goats (**a** pigs; **b** sheep and goats).

The prevalence of *E*. *bieneusi* in pigs varied from 16.4% in Jilin province to 100.0% in Inner Mongolia Autonomous Region (Supplementary Data Table 1). The overall prevalence of *E*. *bieneusi* acquired by using the random effects model in meta-analysis was 45.1% (95% *CI*: 0.227–0.674, *I*^2^ = 98.183%, *p *< 0.0001, Fig. 4a). A total of 38 genotypes of *E*. *bieneusi* were detected in pigs, including genotype CHN7, O, EbpC, D, EbpA, EbpD, Henan-IV, CS-1, CS-2, CS-3, CS-4, CS-5, CS-6, CS-7, CS-8, H, LW1, CHG19, CHC5, SC02, WildBoar 10, WildBoar 8, WildBoar 9, WildBoar 7, PigEBITS5, WildBoar 11, RWSH4, EbpB, EBITS3, G, Henan-I, CS-9, H/EbpCc, Henan-III, CHN1, CHN8, CHN9 and CHN10 (Supplementary Data Table 2).

### Prevalence of *E*. *bieneusi* in sheep and goats

The prevalence of *E*. *bieneusi* in sheep and goats varied from 4.4% in Heilongjiang Province to 69.3% in Inner Mongolia Autonomous (Supplementary Data Table 1). The overall prevalence of *E*. *bieneusi* in sheep and goats acquired by using the random effects model in meta-analysis was 28.1% (95% *CI*: 0.146–0.415, *I*^2^ = 98.716%, *p* < 0.0001, Fig. 4b). A total of 66 genotypes of *E*. *bieneusi* were detected in pigs, including genotype BEB6, CM7, SX1, E, CD6, Peru6, D, O, COS-I, COS-II, COS-III, COS-IV, COS-V, COS-VI, COS-VII, NESH1, NESH2, NESH3, NESH4, NESH5, NESH6, EbpC, EbpA, COG-I, CM4, CHS3, CHS4, CHS5, CHS6, CHS7, CHS8, CHS9, CHS10, CHS11, CHS12, CHS13, CHS14, CHS15, CHS16, CHS17, KIN-1, J, CHG1, CHG2, CHG3, CHG5, CHG6, CHG7, CHG8, CHG9, CHG10, CHG11, CHG12, CHG13, CHG14, CHG16, CHG17, CHG18, CHG19, CHG20, CHG21, CHG22, CHG23, CHG24, CHG25 and CHG5 (Supplementary Data Table 2).

### Prevalence of *E*. *bieneusi* in deer

**Figure 5:**
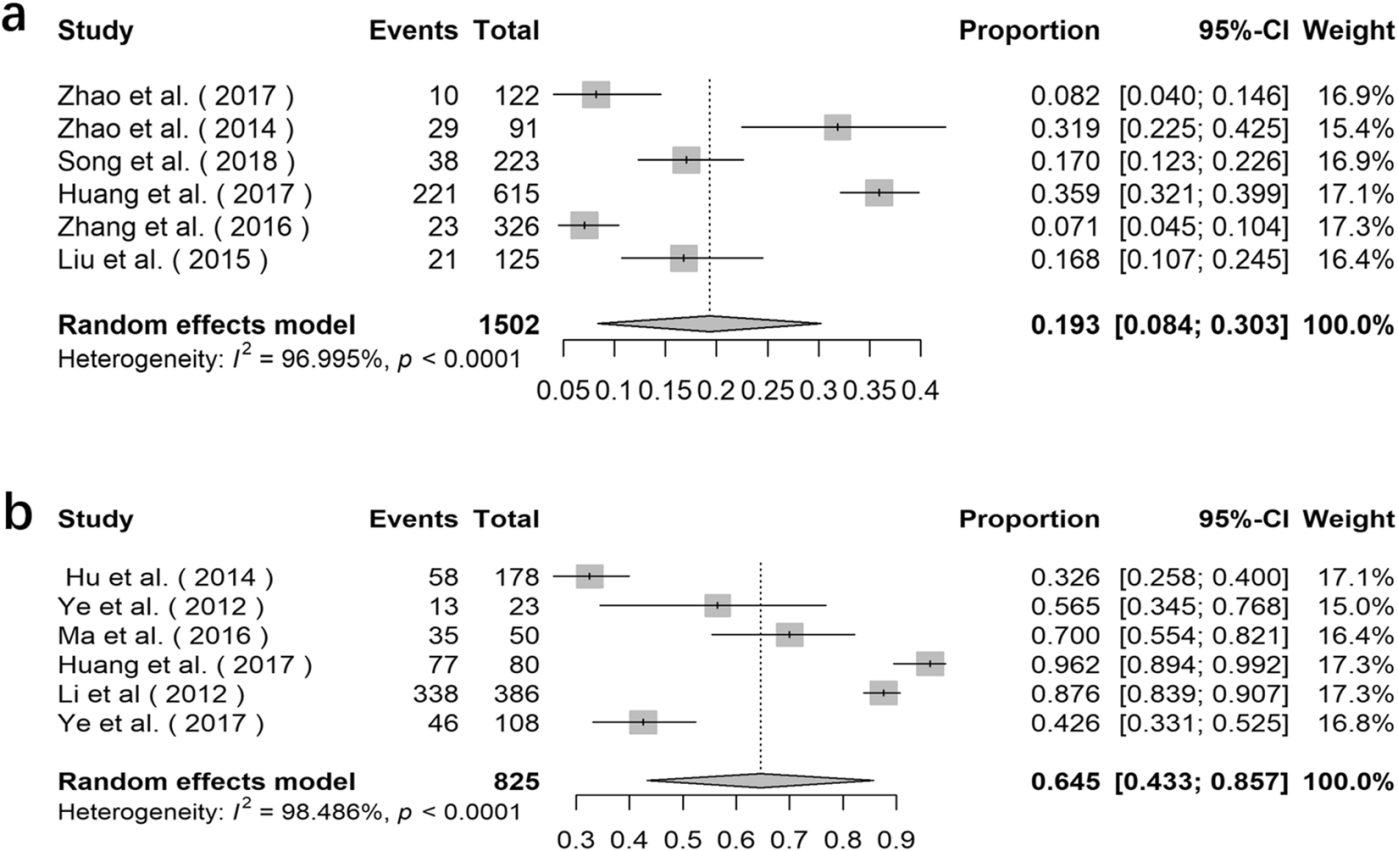
Forest plot diagram showing *E*. *bieneusi* infection in deer and water (**a** deer; **b** water).

The prevalence of *E*. *bieneusi* in deer varied from 6.8% in Heilongjiang Province to 44.1% in Jilin Province (Supplementary Data Table 1). The overall prevalence of *E*. *bieneusi* acquired by using the random effects model in meta-analysis was 19.3% (95% *CI*: 0.084–0.303, *I*^2^ = 96.995%, *p* < 0.0001, Fig. 5a). A total of 39 genotypes of *E*. *bieneusi* were detected in deer, including genotype J, BEB6, EbpC, CHN-DC1, KIN-1, JLD-I, JLD-II, JLD-III, JLD-IV, JLD-V, JLD-VI, JLD-VII, JLD-VIII, JLD-IX, JLD-X, JLD-XI, JLD-XII, JLD-XIII, JLD-XIV, Peru6, CHN-RD1, CHN-RD2, CHN-RD3, CHN-RD4, HLJD-I, HLJD-II, HLJD-III, HLJD-IV, HLJD-V, HLJD-VI, CHS9, SC03, COS-I, EbpA, D, HND-I, HND-II, HND-III and HND-IV (Supplementary Data Table 2).

### Detection rate of *E*. *bieneusi* in water

The detection rate of *E*. *bieneusi* in water varied from 31.5% to 100% (Supplementary Data Table 3), which was prominently higher than in other samples. The overall detection rate of *E*. *bieneusi* in water acquired by using the random effects model in meta-analysis was 64.5% (95% *CI*: 0.433–0.857,* I*^2^ = 98.486%, *p* < 0.0001, Fig. 5b). Totally, 49 genotypes of *E*. *bieneusi* were detected in water, including genotype EbpA, EbpB, EbpC, EbpD, I, J, C, D, CS-8, PtEb IV, PtEbIX, Peru6, Peru8, Peru 11, PigEBITS4, PigEBITS5, PigEBITS7, PigEBITS8, G, O, WL4, WL12, WL14, WL15, Type IV, LW1d, ESH-01, ESH-02, ESH-03, ESH-04, ESH-05, Henan V, SHW2, SHW1, BEB6, WW1, WW2, WW3, WW4, WW5, WW6, WW7, WW8, WW9, HNWW1, HNWW2, HNWW3, HNWW4 and HNWW5 (Supplementary Data Table 3).

### Species, genotypes and distribution of microsporidia

**Figure 6:**
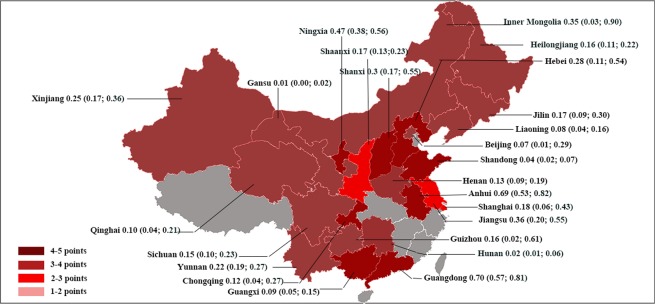
The prevalence of *E*. *bieneusi* in animals in China.

Currently, only three species of microsporidia were confirmed to be existed in China, including *E*. *bieneusi*, *Nosema* and *E*. *cuniculi*. Prevalence of other species is still unclear. So far, 221 genotypes of *E*. *bieneusi* (Supplementary Data Table 2) and 1 genotype of *E*. *cuniculi* (genotype PTP1) were detected in China. The most prevalent genotype of *E*. *bieneusi* was genotype D, followed by BEB6 and EbpC. For *Nosema*, 6 *Nosema* types were detected in China, including *Nosema A*, *Nosema B*, *Nosema C*, *Nosema D*, *Nosema bombi*, and *Nosema ceranae* (Supplementary Data Table 4). Studies on human infection of *E*. *bieneusi* were rare, only six provinces, one autonomous region and one municipality had reported human infection of *E*. *bieneusi* (Table 1). Like humans, studies on water were limited in 5 provinces and one municipality (Supplementary Data Table 3). However, studies on animal infection were more sufficient compared with those on humans and water, as it covered 17 provinces, 4 autonomous regions and 3 municipalities (Fig. 6).

## Discussion

Our aim was to conclude the prevalence of microsporidia in China, and according to our statistics, higher prevalence was observed in animals and water when compared with that in human-beings. For example, the highest infection rate reported in humans was 22.2%, while that was 100% in both animals and water. The poor living condition may be responsible for the high prevalence of animals. As for the high prevalence in water, there were a total of ten records on water, and six of these records focused on waste water. Waste water is a general term for water and runoff rainwater discharged during residents’ activities, which is seriously polluted by feces, domestic garbage and industrial waste. The high prevalence of *E*. *bieneusi* in water may be attributed to the pollution. Moreover, it was reported that water, either consumed directly by drinking or indirectly via irrigating or washing foods, bathing, washing, or for recreation, provided a crucial medium for spore survival and transmission^19^.

Furthermore, our data showed the highest overall estimated prevalence of *E*. *bieneusi* in humans was observed in AIDS patients (8.1%). In nature, there is a balanced interaction between *E*. *bieneusi* and their hosts, which leads to long-term subclinical infections^20^. When the host is immunocompromised, infection can cause overt signs of clinical disease^21^. It was reported that AIDS patients whose CD4 + T cells counting was less than 50 per mm^3^ blood were most likely to experience persistent diarrhea, weight loss, and abdominal pain associated with *E*. *bieneusi* or *E*. *intestinalis* infections. Since the first case of AIDS was reported in 1985, the prevalence of AIDS in China has become increasingly severe. According to a joint assessment by the Chinese Center for Disease Control and Prevention, Joint United Nations Programme on HIV and AIDS and the World Health Organization, by the end of 2018, there will be approximately 1.25 million people living with HIV in China. Such a large number makes us think about how to improve their life quality. Thus, further studies on therapy and prevention are needed as there is no effective solution currently. At the same time, the high prevalence of *E*. *bieneusi* in diarrhea patients should not be ignored as there are approximately 70 million cases suffering from diarrheal diseases annually in China. On the other hand, considering the high prevalence, we suggest clinical doctors taking *E*. *bieneusi* infection into consideration when dealing with diarrheal issue and AIDS patients. Also, for laboratory researchers who need *E*. *bieneusi* strain for further study, feces from AIDS patients and diarrheal patients may be the first choice for separating *E*. *bieneusi strains*.

To our knowledge, this is the first systematic review and meta-analysis of the prevalence of *E*. *bieneusi* in China. However, there is some limitation of this meta-analysis which may influence the results. Firstly, the research on human-beings was insufficient as there were only six provinces, one autonomous region and one municipality have reported the infection of humans. Research on humans still has a lot of blanks waiting for us to fill. Secondly, in order to fully understand the prevalence of *E*. *bieneusi* in animals, more different kinds of animal hosts should be included. Moreover, instead of being confined to several provinces, sampling regions of the same host need to be expanded. Thirdly, repeat detection and negative control were ignored in some studies, which may bring about an inaccurate result. Fourthly, included studies on water mainly focused on waste water, which prevented us from further analysis of other water source, such as combined sewer overflow and drink water. Fifthly, the lack of usable data forced us to give up the analysis of other species of microsporidia and focused only on *E*. *bieneusi*.

In conclusion, this review provides a broad outlook of the prevalence of *E*. *bieneusi* in China, but there are still some problems that need to be solved by all of us. In addition, as chemoprophylaxis and chemotherapeutic treatment modalities are not available, virtually nothing is known about immunity, vaccines are nonexistent, and the study of *E*. *bieneusi* are supposed to be more further improved. Meanwhile, it’s necessary to strengthen the prevention. Effective measurements including protection of water source and food from pollution, environmental hygiene, personal hygiene and health education should be emphasized.

## Materials and Methods

The current study followed the Meta-analysis Of Observational Studies in Epidemiology (MOOSE) guidelines^22^ (Supplementary Data Table 5).

### Search strategy

The article searching was conducted in 8 databases, including both English articles and Chinese articles, and 2 articles came from other sources. Pubmed, Embase, Web of science and Cochrane library were applied for looking up English articles, while Chinese articles searching was conducted in CBM, CNKI, VIP and Wanfang databases. Manual searches were proceeded as a supplement. Time limitation of publication was no later than February 16, 2018. Keywords applied for searching were Microsporidiosis, Microsporidia, *Microsporidium*, Microspora, *Nosema*, *E*. *bieneusi*, *Encephalitozoon* spp., China, Epidemiology, Genotype and Prevalence.

### Study selection

Articles had to meet the following inclusion criteria: all chosen articles should be published up to February 2018; the study should be one of the following types: descriptive study, cross-sectional study, case-control study or epidemiology study; the language of the article was supposed to be either English or Chinese; the studies should be closely related to the prevalence and genotypes of microsporidia.

Articles were excluded if they meet any of the following exclusion criteria: studies that used other diagnostic methods, except staining and molecular techniques; articles written in a language other than English and Chinese; unscientific publication about microsporidia infection (abstracts, national conference proceedings); duplicate studies with overlapping data; articles had poor association with the prevalence and genotypes of microsporidia.

The suitability of all studies was considered by three different authors (L.Q., W.L. and J.P.). Discrepancies were resolved by consensus. After selecting articles, the following information was recorded in a standard data extraction form. A flow diagram of the study design process has been shown in Fig. 1.

### Methodological Quality

The methodological quality of the included studies with an accessible full text was independently assessed by 2 reviewers (W.L. and J.P.) using a modified Newcastle-Ottawa scoring guide^23^. To assess the quality of included studies, the scoring approach was applied. One point each were given to the studies if they comply with the following scoring guidelines: I Population contained a mixture of specialties at multiple sites; II Large sample sizes (no less than 200); III Comparability between respondent and non-respondent characteristics was established, and the response rate was satisfactory; IV Repeat detection; V Reported descriptive statistics to describe the population (e.g., age, sex) with proper measures of dispersion (e.g., standard deviation, standard error, range). Up to five points could be assigned to each study (low quality: 0~2 points; moderate quality: 3 points; high quality: 4~5 points). Discrepancies were resolved by consensus or arbitrated by a third reviewer (W.X.). The results were demonstrated in Tables 1, 2.

### Data extraction and analysis

After study selection, the following data were extracted by three authors (L.Q., W.X. and W.L.) independently from included articles: hosts, province, year of publication, total participants, positive cases, detection method, genus, gender and immune function status. Considering the close relationship between *E*. *bieneusi* and HIV/AIDS patients with diarrhea, which is the most typical symptom, individuals were divided into four groups: including diarrhea patients, HIV/AIDS patients, other patients and healthy people. Patients with both diarrhea and HIV positive were divided into HIV/AIDS group. The group of other patients was composed of cancer patients, outpatients and in-hospital patients. Healthy people group included control group of case-control studies and students. Fact estimates and 95% CI of prevalence of all involved studies were assessed. The random-effects model and subgroup analysis were conducted to investigate the potential source of high heterogeneity. The total prevalence and group-specific prevalence were considered by gender (male and female), sampling region and stool appearance. Forest plots were used to show the heterogeneity among the studies, which showed proportions of individual studies and total prevalence. The meta-analysis was completed with R3.4.3. Distribution map of animal infection rate was drawn with Photo-shop, and the maps of humans and water were omitted for the lack of data.

## Supplementary information

Dataset 1

## Data Availability

All data generated or analyzed during this study are included in this published article and its Supplementary Information.
