# Author Correction: The prevalence of microsporidia in China: A systematic review and meta-analysis

**DOI:** 10.1038/s41598-019-54376-8

**Published:** 2019-12-06

**Authors:** Luyao Qiu, Wanyuan Xia, Wendao Li, Jing Ping, Songtao Ding, Handeng Liu

**Affiliations:** 1 Department of Cell Biology and Genetics, Experimental Teaching Center, Chongqing Medical University, Chongqing, 400016 P.R. China; 2 College of Pediatrics, Chongqing Medical University, Chongqing, 401331 P.R. China; 3 Department of Health Statistics, School of Public Health and Management, Chongqing Medical University, Chongqing, 400016 P.R. China

Correction to: *Scientific Reports* 10.1038/s41598-019-39290-3, published online 28 February 2019

In the original version of this Article, Luyao Qiu and Wanyuan Xia were omitted as equally contributing authors. This has now been corrected in the PDF and HTML versions of the paper.

