# Supplementary material for: The prevalence of microsporidia in China : A systematic review and meta-analysis
Source: Sci Rep. 2019 Feb 28;9:3174. doi: 10.1038/s41598-019-39290-3 (PMC6395699; doi:10.1038/s41598-019-39290-3)
Supplement: Supplementary file 1 — Dataset 1 [file 41598_2019_39290_MOESM1_ESM.pdf]

The prevalence of microsporidia in China： A systematic review and meta-analysis

Luyao Qiu[1,2], Wanyuan Xia[3], Wendao Li[1,2], Jing Ping[1,2], Songtao Ding[1] , Handeng Liu[1*]

*Corresponding author

* Correspondence to hdliu@cqmu.edu.cn

Author	Year	Animal hosts	Origin	Positive cases	Total	Prevalence	Genus&Species	Score	Type of study	Reference
Zhong et al.	2017	Grey kangaroos	Jiangsu	0	23	0	*E.bieneusi*	2	C-S	[1]
Qi et al.	2015	Pet Chinchillas	Guizhou	0	12	0	*E.bieneusi*	3	C-S	[2]
Yang et al.	2016	Rabbits	Heilongjiang	0	65	0	*E.bieneusi*	3	C-S	[3]
Yu et al.	2017	Monkeys	Beijing	0	33	0	*E.bieneusi*	4	C-S	[4]
Xu et al.	2016	Raccoon dogs	Shandong	0	29	0	*E.bieneusi*	5	C-S	[5]
Li et al.	2011	Bumblebees	Gansu	1	227	0.4	*Nosema*	3	C-S	[6]
Zhang et al.	2016	Rabbits	Liaoning	1	136	0.7	*E.bieneusi*	3	C-S	[7]
Zhang et al.	2016	Rabbits	Jilin	3	290	1.0	*E.bieneusi*	3	C-S	[7]
Ma et al.	2017	Yaks	Gansu	4	353	1.1	*E.bieneusi*	4	C-S	[8]
Xu et al.	2016	Raccoon dogs	Heilongjiang	1	40	2.5	*E.bieneusi*	5	C-S	[5]
Qi et al.	2015	Pet Chinchillas	Beijing	1	26	3.8	*E.bieneusi*	3	C-S	[2]
Qi et al.	2015	Pet Chinchillas	Henan	4	102	3.9	*E.bieneusi*	3	C-S	[2]
Zhao et al.	2015	Raccoon Dogs	Heilongjiang	2	49	4.1	*E.bieneusi*	2	C-S	[9]
Li et al.	2014	Sheep	Heilongjiang	2	45	4.4	*E.bieneusi*	2	C-S	[10]
Karim et al.	2014	Snakes	Guangxi	11	240	4.6	*E.bieneusi*	4	C-S	[11]
Yue et al.	2017	Donkeys	Shandong	11	224	4.9	*E.bieneusi*	4	C-S	[12]
Karim et al.	2014	NHPs	Sichuan	17	304	5.6	*E.bieneusi*	4	C-S	[13]
Li et al.	2015	Cats	Heilongjiang	3	52	5.8	*E.bieneusi*	4	C-S	[14]
Jiang et al.	2015	Cattle	Heilongjiang	32	537	6.0	*E.bieneusi*	3	C-S	[15]
Yue et al.	2017	Donkeys	Jilin	3	48	6.3	*E.bieneusi*	4	C-S	[12]
Zhang et al.	2016	Foxes	Jilin	6	91	6.6	*E.bieneusi*	5	C-S	[16]
Li et al.	2015	Dogs	Heilongjiang	18	267	6.7	*E.bieneusi*	4	C-S	[14]

Supplementary Table S1. Prevalence of microsporidia in animals in China.

Author	Year	Animal host	Origin	Positive cases	Total	Prevalence	Genus&Species	Score	Type of study	Reference
Zhao et al.	2017	Deer	Heilongjiang	3	44	6.8	*E.bieneusi*	3	C-S	[17]
Yue et al.	2017	Donkeys	Liaoning	2	29	6.9	*E.bieneusi*	4	C-S	[12]
Li et al.	2011	Bumblebees	Qinghai	4	57	7.0	*Nosema*	3	C-S	[6]
Ma et al.	2014	Yaks	Qinghai	23	327	7.0	*E.bieneusi*	4	C-S	[18]
Zhang et al.	2016	Deer	Jilin	23	326	7.1	*E.bieneusi*	5	C-S	[19]
Zhang et al.	2018	Yaks	Qinghai	40	554	7.2	*E.bieneusi*	4	C-S	[20]
Li et al.	2011	Bumblebees	Sichuan	46	627	7.3	*Nosema*	3	C-S	[6]
Karim et al.	2013	NHPs	Sichuan	10	135	7.4	*E.bieneusi*	4	C-S	[21]
Zhang et al.	2011	Dogs	Jilin	2	26	7.8	*E.bieneusi*	3	C-S	[22]
Zhang et al.	2016	Foxes	Heilongjiang	6	74	8.1	*E.bieneusi*	5	C-S	[16]
Zhao et al.	2017	Deer	Liaoning	5	60	8.3	*E.bieneusi*	3	C-S	[17]
Karim et al.	2014	NHPs	Guangxi	31	363	8.5	*E.bieneusi*	4	C-S	[13]
Karim et al.	2013	NHPs	Guangxi	31	363	8.5	*E.bieneusi*	4	C-S	[21]
Tian et al.	2015	Pandas	Shannxi	4	46	8.7	*E.bieneusi*	2	C-S	[23]
Karim et al.	2013	NHPs	Henan	14	139	10.1	*E.bieneusi*	4	C-S	[21]
Yang et al.	2015	Raccoon dogs	Heilongjiang	17	162	10.5	*E.bieneusi*	3	C-S	[24]
Zhao et al.	2017	Deer	Liaoning	2	18	11.1	*E.bieneusi*	3	C-S	[17]
Li et al.	2011	Bumblebees	Inner Mongolia	11	98	11.2	*Nosema*	3	C-S	[6]
Karim et al.	2014	Cats	Henan	11	96	11.5	*E.bieneusi*	4	C-S	[25]
Karim et al.	2014	Dogs	Chongqing	4	34	11.8	*E.bieneusi*	4	C-S	[25]
Zhong et al.	2017	NHPs	Sichuan&Guizhou	46	369	12.5	*E.bieneusi*	4	C-S	[26]
Du et al.	2015	NHPs	Shaanxi	25	197	12.7	*E.bieneusi*	2	C-S	[27]

Supplementary Table S1. Prevalence of microsporidia in animals in China.

Author	Year	Animal host	Origin	Positive cases	Total	Prevalence	Genus&Species	Score	Type of study	Reference
Tian et al.	2015	Pandas	Shaanxi	5	36	13.9	*E.bieneusi*	2	C-S	[23]
Jiang et al.	2015	Sheep	Heilongjiang	68	489	13.9	*E.bieneusi*	3	C-S	[15]
Karim et al.	2014	Dogs	Henan	34	244	13.9	*E.bieneusi*	4	C-S	[25]
Yang et al.	2016	Rabbits	Heilongjiang	22	150	14.7	*E.bieneusi*	3	C-S	[3]
Zhao et al.	2015	Golden takins	Shaanxi	28	191	14.7	*E.bieneusi*	2	C-S	[28]
Xu et al.	2016	Raccoon dogs	Liaoning	11	72	15.3	*E.bieneusi*	5	C-S	[5]
Li et al.	2015	Animals in zoo	Henan	32	203	15.8	*E.bieneusi*	4	C-S	[29]
Li et al.	2016	Wildlife	Sichuan	43	272	15.8	*E.bieneusi*	3	C-S	[30]
Peng et al.	2016	Cashmere goats	Shaanxi	50	315	15.9	*E.bieneusi*	3	C-S	[31]
Deng et al.	2016	Horses	Sichuan	25	156	16.0	*E.bieneusi*	4	C-S	[32]
Zhao et al.	2015	Blue Foxes	Jilin	6	37	16.2	*E.bieneusi*	2	C-S	[9]
Zhao et al.	2015	Blue Foxes	Heilongjiang	12	73	16.4	*E.bieneusi*	2	C-S	[9]
Zhang et al.	2011	Pigs	Jilin	10	61	16.4	*E.bieneusi*	3	C-S	[22]
Qi et al.	2016	Dairy calves	Xinjiang	85	514	16.5	*E.bieneusi*	4	C-S	[33]
Deng et al.	2016	Squirrels	Sichuan	24	144	16.7	*E.bieneusi*	3	C-S	[34]
Liu et al.	2015	Reindeer	Heilongjiang	21	125	16.8	*E.bieneusi*	3	C-S	[35]
Song et al.	2018	Deer	Sichuan	38	223	17.0	*E.bieneusi*	4	C-S	[36]
Deng et al.	2018	Pet chipmunks	Sichuan	49	279	17.6	*E.bieneusi*	4	C-S	[37]
Zhang et al.	2016	Foxes	Hebei	25	141	17.7	*E.bieneusi*	5	C-S	[16]
Yang et al.	2017	NHPs	Beijing	37	205	18.0	*E.bieneusi*	3	C-S	[38]

Supplementary Table S1. Prevalence of microsporidia in animals in China.

Author	Year	Animal host	Origin	Positive cases	Total	Prevalence	Genus&Species	Score	Type of study	Reference
Ye et al.	2013	NHPs	Guangxi	38	205	18.5	*E.bieneusi*	4	C-S	[39]
Wang et al.	2018	Rabbits	Henan	235	1213	19.4	*E.cuniculi*	5	C-S	[40]
Wang et al.	2016	Calves	Shaanxi	73	371	19.7	*E.bieneusi*	4	C-S	[41]
Wu et al.	2018	Black bears	Yunnan	80	405	19.8	*E.bieneusi*	5	C-S	[42]
Zhao et al.	2014	Deer	Heilongjiang	1	5	20.0	*E.bieneusi*	3	C-S	[43]
Karim et al.	2014	Dogs	Shaanxi	6	30	20.0	*E.bieneusi*	4	C-S	[25]
Chen et al.	2010	Bumblebees	Gansu&Qinghai&Sichuan&Inner Mongolia	210	1008	20.8	*Nosema*	4	C-S	[44]
Li et al.	2016	Dairy cattle	Henan	163	770	21.2	*E.bieneusi*	5	C-S	[45]
Karim et al.	2013	NHPs	Yunnan	31	145	21.4	*E.bieneusi*	4	C-S	[21]
Karim et al.	2014	NHPs	Yunnan	31	144	21.5	*E.bieneusi*	4	C-S	[13]
Zhao et al.	2015	Goats	Heilongjiang	12	55	21.8	*E.bieneusi*	2	C-S	[46]
Zhao et al.	2016	Birds	Heilongjiang	43	194	22.2	*E.bieneusi*	2	C-S	[47]
Zhao et al.	2015	Sheep	Heilongjiang	31	138	22.5	*E.bieneusi*	2	C-S	[46]
Zhang et al.	2018	Sheep	Qinghai	73	312	23.4	*E.bieneusi*	4	C-S	[20]
Zhao et al.	2014	Deer	Heilongjiang	13	52	25.0	*E.bieneusi*	3	C-S	[43]
Karim et al.	2014	Dogs	Sichuan	10	40	25.0	*E.bieneusi*	4	C-S	[25]
Tang et al.	2018	Calves	Shanghai	214	809	26.5	*E.bieneusi*	5	C-S	[48]
Deng et al.	2017	Bears	Sichuan&Guizhou	29	106	27.4	*E.bieneusi*	4	C-S	[49]
Yang et al.	2015	Foxes	Heilongjiang	53	191	27.7	*E.bieneusi*	3	C-S	[24]
Ye et al.	2012	Monkeys	Guizhou	116	411	28.2	*E.bieneusi*	4	C-S	[50]
Deng et al.	2016	Horses	Yunnan	50	177	28.2	*E.bieneusi*	4	C-S	[32]

Supplementary Table S1. Prevalence of microsporidia in animals in China.

Author	Year	Animal host	Origin	Positive cases	Total	Prevalence	Genus&Species	Score	Type of study	Reference
Shi et al.	2016	Goats	Henan & Yunnan &Anhui&Chongqing&Shaanxi	176	611	28.8	*E.bieneusi*	4	C-S	[51]
Karim et al.	2015	NHPs	Hebei&Hubei&Shanxi&Hunan&Beijing&Shanghai	148	496	29.8	*E.bieneusi*	5	C-S	[52]
Qi et al.	2018	Camels	Xinjiang	122	407	30.0	*E.bieneusi*	4	C-S	[53]
Zhao et al.	2015	Dairy Cattle	Heilongjiang	40	133	30.1	*E.bieneusi*	3	C-S	[54]
Xu et al.	2016	Raccoon dogs	Jilin	34	110	30.9	*E.bieneusi*	5	C-S	[5]
Qi et al.	2016	Horses	Xinjiang	81	262	30.9	*E.bieneusi*	4	C-S	[55]
Peng et al.	2016	Dairy goats	Shaanxi	56	170	32.9	*E.bieneusi*	3	C-S	[31]
Yu et al.	2017	Monkeys	Shanxi	7	21	33.3	*E.bieneusi*	4	C-S	[4]
Shi et al.	2016	Sheep	Liaoning&Heilongjiang&Henan	177	414	42.8	*E.bieneusi*	4	C-S	[51]
Li et al.	2018	Pandas	Sichuan	69	200	34.5	*E.bieneusi*	4	C-S	[56]
Huang et al.	2017	Deer	Henan &Jilin	221	615	35.9	*E.bieneusi*	3	C-S	[57]
Zhong et al.	2017	Red kangaroos	Jiangsu	14	38	36.8	*E.bieneusi*	2	C-S	[1]
Zhang et al.	2011	Cattle	Jilin	35	93	37.6	*E.bieneusi*	3	C-S	[22]
Li et al.	2014	Pigs	Jilin	10	25	40	*E.bieneusi*	3	C-S	[58]
Xu et al.	2016	Raccoon dogs	Hebei	22	54	40.7	*E.bieneusi*	5	C-S	[5]
Li et al.	2017	Wild boars	Sichuan	147	357	41.2	*E.bieneusi*	4	C-S	[59]
Zhao et al.	2014	Deer	Jilin	15	34	44.1	*E.bieneusi*	3	C-S	[43]

Supplementary Table S1. Prevalence of microsporidia in animals in China.

Author	Year	Animal host	Origin	Positive cases	Total	Prevalence	Genus&Species	Score	Type of study	Reference
Li et al.	2014	Pigs	Heilongjiang	39	86	45.3	*E.bieneusi*	3	C-S	58
Zhao et al.	2017	Eriocheir sinensis	Jiangsu	449	990	45.4	-	4	C-S	60
Li et al.	2016	Dairy cattle	Ningxia	51	109	46.8	*E.bieneusi*	5	C-S	45
Peng et al.	2016	Meat goats	Henan	73	144	50.7	*E.bieneusi*	3	C-S	31
Yu et al.	2017	Monkeys	Shanghai	42	70	60.0	*E.bieneusi*	4	C-S	4
Li et al.	2014	Pigs	Jilin	94	156	60.3	*E.bieneusi*	2	C-S	10
Ye et al.	2015	Sheep	Inner Mongolia	260	375	69.3	*E.bieneusi*	4	C-S	61
Yu et al.	2017	Monkeys	Anhui	25	36	69.4	*E.bieneusi*	4	C-S	4
Karim et al.	2014	NHPs	Guangdong	40	57	70.2	*E.bieneusi*	4	C-S	13
Zhao et al.	2014	Pigs	Heilongjiang	85	95	89.5	*E.bieneusi*	3	C-S	62
Li et al.	2014	Pigs	Inner Mongolia	2	2	100.0	*E.bieneusi*	3	C-S	58
Li et al.	2014	Pigs	Tianjin&Jilin&Heilongjiang&Liaoning&Inner Mongolia	11	64	17.2	*E.bieneusi*	3	C-S	63
Karim et al.	2014	NHPs	Henan	39	518	7.5	*E.bieneusi*	4	C-S	13
Karim et al.	2014	NHPs	Sichuan	17	304	5.6	*E.bieneusi*	4	C-S	13
Ma et al.	2015	Dairy Cattle	Shandong	3	148	2.0	*E.bieneusi*	3	C-S	64
Ma et al.	2015	Dairy Cattle	Henan	19	298	6.4	*E.bieneusi*	3	C-S	64
Ma et al.	2015	Beeves	Henan	9	166	5.4	*E.bieneusi*	3	C-S	64
Ma et al.	2015	Buffalos	Hunan	4	181	2.2	*E.bieneusi*	3	C-S	64
Hu et al.	2017	Dairy cattle	Tianjin&Hebei	202	1040	19.4	*E.bieneusi*	3	C-S	65
Xu et al.	2016	Cats	Shanghai	9	160	5.6	*E.bieneusi*	3	C-S	66

Supplementary Table S1. Prevalence of microsporidia in animals in China.

Author	Year	Animal host	Origin	Positive cases	Total	Prevalence	Genus&Species	Score	Type of study	Reference
Xu et al.	2016	Dogs	Shanghai	29	485	6.0	*E.bieneusi*	3	C-S	[66]

Supplementary Table S1. Prevalence of microsporidia in animals in China.

Origin	Host	Genus&Species	Total of genotypes	Genotypes	Reference
Jilin	Donkeys	*E.bieneusi*	2	D,NCD-1	[12]
Jilin	Deer	*E.bieneusi*	22	J,BEB6,EbpC,CHN-DC1,KIN-1,HLJD-V,HLJD-I,JLD-VIII, JLD-IX, JLD-I, JLD-V, JLD-XI,JLD-XIV,JLD-II, JLD-III, JLD-IV, JLD-VI, JLD-VII, JLD-XIII, HLJD-IV, JLD-XII, JLD-X	[19，43，57]
Jilin	Foxes	*E.bieneusi*	4	NCF2,Peru8,Type IV,D	[16]
Jilin	Chicken	*E.bieneusi*	1	CC-1	[63]
Jilin	Pigs	*E.bieneusi*	21	Henan-IV,CS-1,CS-4,CS-6,EbpA,EbpB,EbpC,EbpD,EBITS3,G,Henan-I,CS-9,H/EbpCc,Henan-III,O,CS-8,CHN1,CHN7,CHN8,CHN9,CHN10	[10，22，58，63]
Jilin	Cattle	*E.bieneusi*	5	I,J,CHN1,CHN3,CHN4	[22]
Jilin	Dogs	*E.bieneusi*	2	CHN5,CHN6	[22]
Jilin	Diarrheal Children	*E.bieneusi*	6	I,J,CHN1,CHN2,CHN3,CHN4	[22]
Jilin	Foxes	*E.bieneusi*	1	D	[9]
Jilin	Rabbits	*E.bieneusi*	1	D	[7]
Beijing	NHPs	*E.bieneusi*	16	D,Peru8,Peru11,CM1,CM2,CC4, WL21,Type IV,O, EbpA, EbpC, EbpD, PigEBITS5,CM10,CM11,CS-1	[38，52]
Beijing	Pet chinchillas	*E.bieneusi*	1	BEB6	[2]
Shandong	Donkeys	*E.bieneusi*	3	D,J,NCD-2	[12]
Shandong	Dairy cattle	*E.bieneusi*	2	I,J	[64]
Shandong	Pandas	*E.bieneusi*	1	SC02	[56]
Liaoning	Donkeys	*E.bieneusi*	2	D,J	[12]
Liaoning	Rabbits	*E.bieneusi*	1	D	[7]

Supplementary Table S2. Genotypes of *E.bieneusi* detected in human and animal hosts in China

Origin	Host	Genus&Species	Total of genotypes	Genotypes	Reference
Liaoning	Sheep	*E.bieneusi*	1	BEB6	51
Inner Mongolia	Pigs	*E.bieneusi*	3	CHN7,O,EbpC	63
Inner Mongolia	Sheep	*E.bieneusi*	2	BEB6,CM7	61
Shaanxi	Dogs	*E.bieneusi*	4	PtEbIX,EbpC,CD8,CD9	25
Shaanxi	NHPs	*E.bieneusi*	5	D,BEB6,MH,XH,BSH	27
Shaanxi	Pandas	*E.bieneusi*	2	EbpC,I-like	23
Shaanxi	Golden takins	*E.bieneusi*	7	BEB6,D,I,TEB1,TEB2,TEB3,TEB4	28
Shaanxi	Goats	*E.bieneusi*	12	BEB6,CHG1,CHG2,SX1,E, F, CHG3, CD6,CHG5, CHG14, CHG16, CHG24	31，51
Shaanxi	Calves	*E.bieneusi*	5	I, J,CHN1,CSX1,CSX2	41
Guangxi	AIDS patients	*E.bieneusi*	7	PigEBITS7,Type IV/K, D,Ebpc,GX25, GX456,GX458	67
Guangxi	NHPs	*E.bieneusi*	9	D,CM1, Peru8, CM2,Peru11,Macaque3a, Macaque4a,WL15,Type IV	13，21，39
Guangxi	Snakes	*E.cuniculi*	6	type IV,Henan V,CRep-1,CRep-2,CRep-3,CRep-4	11
Guizhou	Monkeys	*E.bieneusi*	6	Peru11,WL15,EbpC,TypeIV,Macaque1,Macaque2	50
Guizhou	NHPs	*E.bieneusi*	2	PigEBITS7,D	26
Guizhou	Bears	*E.bieneusi*	3	CHB1,horse2,ABB2	49
Guangdong	NHPs	*E.bieneusi*	7	IV, CM1, Peru8, CM2, D, Peru11, CM3	13，21
Chongqing	Dogs	*E.bieneusi*	2	PtEbIX,CD8	25
Chongqing	Goats	*E.bieneusi*	4	CHG1, CHG3, CD6, CHG12	51
Heilongjiang	Cancer patients	*E.biensusi*	2	D, HLJ-CP1	68
Heilongjiang	Foxes	*E.bieneusi*	4	Peru8, Type IV,CHN-DC1, NCF2	16

Supplementary Table S2. Genotypes of *E.bieneusi* detected in human and animal hosts in China

Origin	Host	Genus&Species	Total of genotypes	Genotypes	Reference
Heilongjiang	Deer	*E.bieneusi*	12	Peru6,CHN-RD1,CHN-RD2,CHN-RD3,CHN-RD4,HLJD-VI, BEB6,HLJD-I, HLJD-II, HLJD-III, HLJD-IV, HLJD-V	17，35，43
Heilongjiang	Chichken	*E.biensusi*	1	Henan-IV	63
Heilongjiang	Pigs	*E.biensusi*	17	EbpC,D,EbpA,EbpD,Henan-IV,O,CS-1,CS-2,CS-3,CS-4,CS-5,CS-6,CS-7,CS-8,D,H,LW1	58，62，63
Heilongjiang	Children	*E.bieneusi*	8	CS-4,EbpC,Henan-IV,NEC1,NEC2,NEC3,NEC4,NEC5	69
Heilongjiang	Sheep	*E.bieneusi*	21	BEB6,Peru6,D,O,COS-I,COS-II,COS-III,COS-IV,COS-V,COS-VI,COS-VII,NESH1,NESH2,NESH3,NESH4,NESH5,NESH6, CHS7, CHS8, CHS9,CHS11	10，15，46，51
Heilongjiang	Goats	*E.bieneusi*	6	BEB6,Peru6, D, EbpC, EbpA, COG-I	46
Heilongjiang	Birds	*E.bieneusi*	7	Peru6,BEB6,D,EbpA,CHN-B1,CHN-B2,CHN-B3	47
Heilongjiang	Foxes	*E.bieneusi*	3	D,CHN-F1, EbpC	9，24
Heilongjiang	Raccoon dogs	*E.biensusi*	4	D, CHN-R1,CHN-DC1,WildBoar3	9，24
Heilongjiang	Rabbits	*E.bieneusi*	8	CHN-RD1,D,Type IV,Peru6,I,CHN-RR1,CHN-RR2,CHN-RR3	3
Heilongjiang	Cattle	*E.bieneusi*	11	NECA1,NECA2,NECA3,NECA4,NECA5,O,I,J,D,EbpA,BEB4	15，54
Heilongjiang	Cats	*E.bieneusi*	2	D,Type IV	14
Heilongjiang	Dogs	*E.bieneusi*	7	D, EbpC,PtEb IX,NED1,NED2,NED3,NED4	14
Sichuan	Horses	*E.biensusi*	6	SC02,horse1,SCH1,SCH2,SCH3,SCH4	32
Sichuan	Dogs	*E.biensusi*	2	PtEbIX,CD1	25
Sichuan	NHPs	*E.biensusi*	8	CM1,BEB6,D,PigEBITS7,CM4,Type IV,SCM01, SC02	13，21，26
Sichuan	Squirrels	*E.bieneusi*	5	D,EbpC,SC02,CE01,CE02	34
Sichuan	Bears	*E.bieneusi*	4	CHB1,SC02,SC01,ABB1	30，49
Sichuan	Pandas	*E.bieneusi*	13	SC02,D,SC04,SC01,SC05,CHB1,F,EbpC,SC06,SC07,SC08,F,Peru 6	30，56

Supplementary Table S2. Genotypes of *E.bieneusi* detected in human and animal hosts in China

Origin	Host	Genus&Species	Total of genotypes	Genotypes	Reference
Sichuan	African lions	*E.bieneusi*	1	D	[30]
Sichuan	Cats	*E.bieneusi*	1	D	[30]
Sichuan	Deer	*E.bieneusi*	3	BEB6,CHS9,SC03	[30、36]
Sichuan	Alpaca	*E.bieneusi*	1	BEB6	[30]
Sichuan	Blackbucks	*E.bieneusi*	1	CHS9	[30]
Sichuan	Monkeys	*E.bieneusi*	1	D	[30]
Sichuan	Boars	*E.bieneusi*	13	EbpC,CHG19,CHC5,F,SC02,WildBoar 10,WildBoar 8,WildBoar 9,WildBoar 7,PigEBITS5,WildBoar 11,D, RWSH4	[59]
Sichuan	Chipmunks	*E.bieneusi*	8	D, Nig7,CHG9,CHY1,SCC-1,SCC-2,SCC-3,SCC-4	[37]
Sichuan	Olive baboons	*E.bieneusi*	1	D	[30]
Sichuan	Northern raccoons	*E.bieneusi*	2	D,SC02	[30]
Sichuan	Ring-tailed lemurs	*E.bieneusi*	1	CHB1	[30]
Sichuan	Northern white-cheeked gibbons	*E.bieneusi*	1	D	[30]
Yunnan	Horses	*E.biensusi*	6	horse2,horse1,SC02,D,YNH1,YNH2	[32]
Yunnan	NHPs	*E.bieneusi*	4	Type IV, CM1,Peru8, D	[13、21]
Yunnan	Goats	*E.bieneusi*	12	BEB6, E, F, D, COS-I, CD6, CHG1,CHG3, CHG5, CHG16, CHG17, CHG19	[51]
Yunnan	Bears	*E.bieneusi*	13	MJ1 ,MJ2 ,MJ3 .MJ4.MJ5,MJ6,MJ7,MJ8,MJ9,MJ10, MJ11,MJ12,MJ13	[42]
Shanghai	Dogs	*E.bieneusi*	2	PtEb IX,D	[66]

Supplementary Table S2. Genotypes of *E.bieneusi* detected in human and animal hosts in China

Origin	Host	Genus&Species	Total of genotypes	Genotypes	Reference
Shanghai	Cats	*E.bieneusi*	2	Type IV, D	[66]
Shanghai	Calves	*E.bieneusi*	4	J, BEB4, CHN4,CHN15	[48]
Shanghai	NHPs	*E.bieneusi*	11	D,O, EbpA, Henan IV,CM15,CM16,CM4,BEB4,EbpC,CM17,CM18	[52]
Shanghai	Monkeys	*E.bieneusi*	7	D,J,CHG1,CHG14,CM19,CM20	[4]
Shanghai	Pandas	*E.bieneusi*	1	SC02	[56]
Hebei	Diarrheal children	*E.bieneusi*	1	D	[70]
Hebei	Foxes	*E.bieneusi*	10	Peru8,TypeIV,D,NCF1,NCF2,NCF3,NCF4,NCF5,NCF6,NCF7	[16]
Hebei	NHPs	*E.bieneusi*	7	Type IV,Henan-IV,D,EbpC,EbpA,CM1,CM8	[52]
Henan	Cattle	*E.bieneusi*	3	I,J,BEB4	[64]
Henan	Beeves	*E.bieneusi*	3	I,J,BEB4	[64]
Henan	Dogs	*E.bieneusi*	14	PtEbIX,EbpA,O,PigEBITS5,D,CD2,CD3,CD4,CD5,CD6,CD7,CM1,Peru8, type IV	[25]
Henan	Cats	*E.bieneusi*	8	D,I, BEB6, PtEbIX, CC1, CC2, CC3, CC4	[25]
Henan	NHPs	*E.bieneusi*	10	Henan V ,D ,CM4 ,EbpC ,PigEBITS7 , IV, I ,CM5 , CM6,CM7	[13]
Henan	HIV positive individuals	*E.bieneusi*	11	EbpD,Peru8,Henan-I,Henan-II,Henan-III,Henan-IV ,Henan-V,EbpC,D,PigEBITS7,TypeIV	[21，71]
Henan	HIV negative individuals	*E.bieneusi*	4	Peru11,EbpC,D,TypeIV	[71]
Henan	Sheep	*E.bieneusi*	10	BEB6, COS-I, CM4, CHG3, CHS3,CHS4, CHS5, CHS6, CHS10, CHS12	[51]
Henan	Pet chinchillas	*E.bieneusi*	2	BEB6,D	[2]
Henan	Deer	*E.bieneusi*	14	BEB6, HLJDI,EbpC, HND-II,COS-I,EbpA,D,JLD-II,HND-I, JLD-VI, HND-III, HND-IV, JLD-XI,JLD-XIV	[57]

Supplementary Table S2. Genotypes of *E.bieneusi* detected in human and animal hosts in China

Origin	Host	Genus&Species	Total of genotypes	Genotypes	Reference
Henan	Goats	*E.bieneusi*	26	BEB6, E, F, KIN-1, D, J, COS-I,CD6, CHG1, CHG2,CHG3,CHG5,CHG6,CHG7, CHG8, CHG9, CHG10,CHG11, CHG13, CHG18,CHG20, CHG21, CHG22, CHG23, CHG25,CHS7	[31, 51]
Henan	Wildlife	*E.bieneusi*	13	D,Peru8,Type IV,CHALT1,CHP1,J,I,CHG1,CHY1,WL2,CHK1,CHK2,CHB1	[29]
Hunan	Buffalos	*E.bieneusi*	1	CHN11	[64]
Hunan	NHPs	*E.bieneusi*	8	EbpC,D,O,Type IV,BEB6,CM12,CM13,CM14	[52]
Hunan	Pandas	*E.bieneusi*	1	SC02	[56]
Xinjiang	Grazing Horse	*E.bieneusi*	19	BEB6,CHG19,CM6,CM7,CM8,CS-1,CS-4,D,EpbA,EbpC,G,horse1,horse2 ,O,Peru8,XJH1,XJH2,XJH3,XJH4	[55]
Xinjiang	Camels	*E.bieneusi*	14	EbpC, EbpA, Henan-IV, BEB6, CM8, CHG16, O, WL17, CAM1, CAM2, CAM3, CAM4, CAM5, CAM6	[53]
Xinjiang	Dairy calves	*E.bieneusi*	6	BEB4,D,I,J,EbpC,CC4	[33]
Jiangsu	Red Kangaroos	*E.bieneusi*	3	CHK1, CSK1, CSK2	[1]
Gansu	White yaks	*E.bieneusi*	3	I,BEB4,WCY1	[8]
Hubei	NHPs	*E.bieneusi*	3	D,EbpC,BEB6	[52]
Anhui	Monkeys	*E.bieneusi*	7	D,J,CM21	[4]
Anhui	Goats	*E.bieneusi*	3	BEB6, CHG5, CHG3	[51]
Shanxi	Monkeys	*E.bieneusi*	7	J,CM19	[4]
Shanxi	NHPs	*E.bieneusi*	4	D, Henan-IV,CM4,CM9	[52]
Shenzhen	Pandas	*E.bieneusi*	1	SC02	[56]

Supplementary Table S2. Genotypes of *E.bieneusi* detected in human and animal hosts in China

Origin	Host	Genus&Species	Total of genotypes or haplotypes	Genotypes	Reference
Fujian	Pandas	*E.bieneusi*	1	SC02	[56]
Zhejiang	Pandas	*E.bieneusi*	1	SC02	[56]
Qinghai	Sheep	*E.bieneusi*	8	BEB6, COS-I,NESH5,CHS13,CHS14,CHS15,CHS16,CHS17	[20]
Qinghai	Yaks	*E.bieneusi*	10	J,BEB4,BEB6,COS-I,NESH5, CHN13,CHN14,I,CHN11,CHN12	[20]
Unknown	In-hospital Children	*E.bieneusi*	16	SH1,SH2,SH3,SH4,SH5,SH6,SH7,SH8,SH9,SH10,SH11,SH12 ,Peru11,EbpC, EbpA, D,	[72]
Hebei &Tianjin	Dairy Cattle	*E.bieneusi*	9	I,J,N,BEB4,BEB6,Ebpc,CHC6,CHC7,CHC8	[65]
Henan&Ningxia	Dairy cattle	*E.bieneusi*	20	I,J,BEB4,BEB6,BEB8,CD6,CM8,COS-I,EbpA,EbpC,D,H,O,CHC1,CHC2,CHC3,CHC4,CHC5,CHG2,CHG3	[45]
Shandong&Liaoning&Heilongjiang&Hebei&Jilin	Raccoon Dogs	*E.bieneusi*	6	D,CHN-DC1, NCF2,CHN-F1,NCR1， NCR2	[5]

Supplementary Table S2. Genotypes of *E.bieneusi* detected in human and animal hosts in China

Origin	Year	Province	Total Samples	Positive（%）	Genus& species	Genotypes	Reference
Huangpu River	2014	Shanghai	178	31.5%(58/178)	*E.bieneusi*	EbpC,EbpA,D,CS-8,PtEbIX,Peru8, Peru 11, PigEBITS4, EbpB, G, O	[73]
Unknown	2012	Guizhou	23	56.5%(13/23)	*E.bieneusi*	Peru11, WL15 , EbpC, Type IV , LW1d	[50]
Waste-water	2016	Shanghai	50	70%(35/50)	*E.bieneusi*	D,ESH-01,ESH-02,ESH-03,ESH-04,ESH-05,EbpA,EbpC, PigEBITS7,Peru 11, Peru 8	[74]
Combined sewer overflow (CSO)	2017	Shanghai	40	92.5%(37/40)	*E.bieneusi*	D, PigEBITS7, Henan V, type IV, Peru 8, Peru 11, SHW2	[75]
Waste-water	2017	Shanghai	40	100%(40/40)	*E.bieneusi*	D, PigEBITS7, Henan V, type IV, Peru 8, Peru 11, SHW1, SHW2	[75]
Waste-water	2012	Shanghai	90	94.5%(85/90)	*E.bieneusi*	Type IV,EbpC,Peru8,Peru11,D,PigEBITS7,PigEBITS8	[76]
Waste-water	2012	Jiangsu	87	62.1%(54/87)	*E.bieneusi*	Peru6,Peru8,Peru11,D,WL12,WL14	[76]
Waste-water	2012	Shandong	109	91.7%(100/109)	*E.bieneusi*	Type IV,EbpC,EbpD,Peru6,D,PtEb IV,BEB6,PigEBITS7, WW1,WW2,WW3,WW4,WW5,WL4,WW6,WW7,PtEb IX,WW8,WW9	[76]
Waste-water	2012	Hubei	100	99.0%(99/100)	*E.bieneusi*	Type IV,EbpC,Peru6,Peru8,Peru11,C,D,EbpA,BEB6, WW3,WL4,PtEb IX	[76]
Waste-water	2017	Henan	108	42.6%(46/108)	*E.bieneusi*	D, BEB6, I, J, PigEbIX, PigEBITS5, EbpA,Peru6, Peru8, Type IV,HNWW1, HNWW2, HNWW3, HNWW4, HNWW5	[77]

Supplementary Table S3. Genotype distribution of *E.bieneusi* detected in water in China.

Region	Host	Genus&Species	Total of haplotypes	Haplotypes	Reference
Qinghai	Bumblebees	*Nosema C*	1	MY227.2	[6]
Qinghai	Bumblebees	*Nosema ceranae*	2	HTES26.1,HTES26.3	[6]
Qinghai	Bumblebees	*Nosema* spp.	2	MY241.1,MY235.2	[6]
Gansu	Bumblebees	*Nosema bombi*	1	QL293.3	[6]
Sichuan	Bumblebees	*Nosema A*	3	LS554.3,LS566.3,LS577.1	[6]
Sichuan	Bumblebees	*Nosema B*	15	GX541.1,GX541.3,GX543.2,LS544.1,LS553.1,LS575.1,LS578.1,MG500.1,MG500.2,MG506.1,MG518.1,MG519.2,MG519.3,ML759.2,ML759.3	[6]
Sichuan	Bumblebees	*Nosema bombi*	7	KD722.2,KD722.3,MG487.2,MG487.5,MG494.2,MG497.3,QL293.3	[6]
Sichuan	Bumblebees	*Nosema C*	8	GX542.1,GX542.2,KD725.1,KD725.2,KD725.3,KD951.1,MG520.1,MZL82.3	[6]
Sichuan	Bumblebees	*Nosema ceranae*	9	KD719.2,KD945.1,MG401.1,MG401.2,MG401.3,MG402.1,MG402.2,MG444.1,MZL76.6	[6]
Sichuan	Bumblebees	*Nosema D*	11	GX538.3,GX540.1,GX540.2,LS546.1,LS546.2,MG491.2,MG491.4,ML913.3,MG525.3,ML916.2,ML916.3	[6]
Sichuan	Bumblebees	*Nosema* spp.	2	LS544.3,MG524.2	[6]
Inner Mongolia	Bumblebees	*Nosema B*	1	ML759.3	[6]
Inner Mongolia	Bumblebees	*Nosema C*	12	ARQ94.2,ARQ94.3,MZL64.2,MZL64.3,MZL66.1,MZL69.1,MZL69.3,MZL82.2,MZL82.3,MZL85.1,MZL85.3,MZL85.6	[6]
Inner Mongolia	Bumblebees	*Nosema ceranae*	4	MZL76.4,MZL76.6,MZL84.2,MZL84.3	[6]
Inner Mongolia	Bumblebees	*Nosema bombi*	3	ARQ86.1,MG487.5,QL293.3	[6]

Supplementary Table S4. Haplotypes of Nosema detected in China.

Supplementary Table S5. MOOSE Checklist for Meta-analyses of Observational Studies

Item No	Recommendation	Reported on Page No
Reporting of background should include		
1	Problem definition	2-5
2	Hypothesis statement	2-5
3	Description of study outcome(s)	2-5
4	Type of exposure or intervention used	N/A
5	Type of study designs used	2-5
6	Study population	2-5
Reporting of search strategy should include		
7	Qualifications of searchers (eg, librarians and investigators)	15-16
8	Search strategy, including time period included in the synthesis and key words	15-16
9	Effort to include all available studies, including contact with authors	15-16
10	Databases and registries searched	15-16
11	Search software used, name and version, including special features used (eg, explosion)	15-16
12	Use of hand searching (eg, reference lists of obtained articles)	15-16
13	List of citations located and those excluded, including justification	15-16
14	Method of addressing articles published in languages other than English	15-16
15	Method of handling abstracts and unpublished studies	15-16
16	Description of any contact with authors	15-16
Reporting of methods should include		
17	Description of relevance or appropriateness of studies assembled for assessing the hypothesis to be tested	15-19
18	Rationale for the selection and coding of data (eg, sound clinical principles or convenience)	15-19
19	Documentation of how data were classified and coded (eg, multiple raters, blinding and interrater reliability)	15-19
20	Assessment of confounding (eg, comparability of cases and controls in studies where appropriate)	15-19
21	Assessment of study quality, including blinding of quality assessors, stratification or regression on possible predictors of study results	15-19
22	Assessment of heterogeneity	15-19
23	Description of statistical methods (eg, complete description of fixed or random effects models, justification of whether the chosen models account for predictors of study results, doseresponse models, or cumulative meta-analysis) in sufficient detail to be replicated	15-19
24	Provision of appropriate tables and graphics	Figure 1-6<br>Table 1-5

Supplementary Table S5: MOOSE Checklist for Meta-analyses of Observational Studies

Item No	Recommendation	Reported on Page No
Reporting of results should include		
25	Graphic summarizing individual study estimates and overall estimate	Figure 2-5
26	Table giving descriptive information for each study included	Table 1-5
27	Results of sensitivity testing (eg, subgroup analysis)	Table 3
28	Indication of statistical uncertainty of findings	5-11
Reporting of discussion should include		
29	Quantitative assessment of bias (eg, publication bias)	N/A
30	Justification for exclusion (eg, exclusion of non-English language citations)	15-16
31	Assessment of quality of included studies	15-17
Reporting of conclusions should include		
32	Consideration of alternative explanations for observed results	11-15
33	Generalization of the conclusions (ie, appropriate for the data presented and within the domain of the literature review)	11-15
34	Guidelines for future research	11-15
35	Disclosure of funding source	11-15

References:

1 Zhong, Z. et al. Molecular characterization and multi-locus genotypes of *Enterocytozoon bieneusi* from captive red kangaroos (Macropus Rfus) in Jiangsu province, China. *Plos One*. 12, (2017).

2 Qi, M. et al. Zoonotic Cryptosporidium spp. and *Enterocytozoon bieneusi* in pet chinchillas (Chinchilla lanigera) in China. *Parasitol Int*. 64, 339-341 (2015).

3 Yang, Z. et al. Subtyping of Cryptosporidium cuniculus and genotyping of *Enterocytozoon bieneusi* in rabbits in two farms in Heilongjiang Province, China. *Parasite*. 23, (2016).

4 Yu, F. et al. High prevalence of *Enterocytozoon bieneusi* zoonotic genotype D in captive golden snub-nosed monkey (Rhinopithecus roxellanae) in zoos in China. *BMC Vet Res*. 13, (2017).

5 Xu, C. et al. Prevalence, risk factors and molecular characterization of *Enterocytozoon bieneusi* in raccoon dogs (Nyctereutes procyonoides) in five provinces of Northern China. *Acta Trop*. 161, 68-72 (2016).

6 Li, J. et al. Diversity of nosema associated with bumblebees (Bombus spp.) from China. *Int J Parasitol*. 42, 49-61 (2012).

7 Zhang, X. X. et al. Molecular characterization of *Enterocytozoon bieneusi* in domestic rabbits (Oryctolagus cuniculus) in northeastern China. *Korean J Parasitol*. 54, 81-85 (2016).

8 Ma, J. et al. Detection of *Enterocytozoon bieneusi* in White Yaks in Gansu Province, China. *Biomed Res Int*. (2017).

9 Zhao, W. et al. Genotyping of *Enterocytozoon bieneusi* in Farmed Blue Foxes (Alopex lagopus) and Raccoon Dogs (Nyctereutes procyonoides) in China. *Plos One*. 10, (2015).

10 Li, W. et al. Genotypes of *Enterocytozoon bieneusi* in livestock in China: High prevalence and zoonotic potential. *Plos One*. 9, (2014).

11 Karim, M. R. et al. First molecular characterization of enteric protozoa and the human pathogenic microsporidian, *Enterocytozoon bieneusi,* in captive snakes in China. *Parasitol Res*. 113, 3041-3048 (2014).

12 Yue, D. M. et al. Occurrence of *Enterocytozoon bieneusi* in donkeys (Equus asinus) in China: A public health concern. *Front Microbiol*. 8, (2017).

13 Karim, M. R. et al. Genetic polymorphism and zoonotic potential of *Enterocytozoon bieneusi* from nonhuman primates in China. *Appl Environ Microb*. 80, 1893-1898 (2014).

14 Li, W. et al. Prevalence and genetic characteristics of Cryptosporidium, *Enterocytozoon bieneusi* and Giardia duodenalis in cats and dogs in Heilongjiang province, China. *Vet Parasitol*. 208, 125-134 (2015).

15 Jiang, Y. et al. Zoonotic and Potentially Host-Adapted *Enterocytozoon bieneusi* Genotypes in Sheep and Cattle in Northeast China and an Increasing Concern about the Zoonotic Importance of Previously Considered Ruminant-Adapted Genotypes. *Appl Environ Microb*. 81, 3326-3335 (2015).

16 Zhang, X. X. et al. Prevalence, risk factors and multilocus genotyping of *Enterocytozoon bieneusi* in farmed foxes (Vulpes lagopus), Northern China. *Parasite Vector*. 9, 72 (2016).

17 Zhao, W., Wang, J., Yang, Z. & Liu, A. Dominance of the *Enterocytozoon bieneusi* genotype BEB6 in red deer (Cervus elaphus) and Siberian roe deer (Capreolus pygargus) in China and a brief literature review. *Parasite*. 24, (2017).

18 Ma, J. et al. *Enterocytozoon bieneusi* Genotypes in Yaks (Bos grunniens) and Their Public Health Potential. *J Eukaryot Microbiol*. 62, 21-25 (2015).

19 Zhang, X. X. et al. Prevalence and genotypes of *Enterocytozoon bieneusi* in sika deer in Jilin

province, Northeastern China. *Acta Parasitol*. 61, 382-388 (2016).

20 Zhang, Q. et al. *Enterocytozoon bieneusi* genotypes in Tibetan sheep and yaks. *Parasitol Res*. 117, 721-727 (2018).

21 Karim, M. R. et al. Multilocus sequence typing of *Enterocytozoon bieneusi* in nonhuman primates in China. *Vet Parasitol*. 200, 13-23 (2014).

22 Zhang, X. et al. Identification and genotyping of *Enterocytozoon bieneusi* in China. *J Clin Microbiol*. 49, 2006-2008 (2011).

23 Tian, G. R. et al. First report of *Enterocytozoon bieneusi* from giant pandas (Ailuropoda melanoleuca) and red pandas (Ailurus fulgens) in China. *Infect Genet Evol*. 34, 32-35 (2015).

24 Yang, Y. et al. Widespread presence of human-pathogenic *Enterocytozoon bieneusi* genotype D in farmed foxes (Vulpes vulpes) and raccoon dogs (Nyctereutes procyonoides) in China: first identification and zoonotic concern. *Parasitol Res*. 114, 4341-4348 (2015).

25 Karim, M. R. et al. Genetic diversity in *Enterocytozoon bieneusi* isolates from dogs and cats in China: Host specificity and public health implications. *J Clin Microbiol*. 52, 3297-3302 (2014).

26 Zhong, Z. et al. Multilocus genotyping of *Enterocytozoon bieneusi* derived from nonhuman primates in southwest China. *Plos One*. 12, (2017).

27 Du S., Z. et al. Cryptosporidium spp., *Giardia intestinalis,* and *Enterocytozoon bieneusi* in captive non-human primates in Qinling mountains. *Korean J Parasitol*. 53, 395-402 (2015).

28 Zhao, G. H. et al. First report of zoonotic Cryptosporidium spp., *Giardia intestinalis* and *Enterocytozoon bieneusi* in golden takins (Budorcas taxicolor bedfordi). *Infect Genet Evol*. 34, 394-401 (2015).

29 Li, J. et al. Molecular Characterization of Cryptosporidium spp., *Giardia duodenalis*, and *Enterocytozoon bieneusi* in Captive Wildlife at Zhengzhou Zoo, China. *J Eukaryot Microbiol*. 62, 833-839 (2015).

30 Li, W. et al. Multilocus genotypes and broad host-range of *Enterocytozoon bieneusi* in captive wildlife at zoological gardens in China. *Parasite Vector*. 9, (2016).

31 Peng, X. Q. et al. Infection rate of *Giardia duodenalis*, Cryptosporidium spp. and *Enterocytozoon bieneusi* in cashmere, dairy and meat goats in China. *Infect Genet Evol*. 41, 26-31 (2016).

32 Deng, L. et al. Molecular characterization and multilocus genotypes of *Enterocytozoon bieneusi* among horses in southwestern China. *ParasiteA Vector*. 9, 561 (2016).

33 Qi, M. et al. Dominance of *Enterocytozoon bieneusi* genotype J in dairy calves in Xinjiang, Northwest China. *Parasitol Int*. 66, 960-963 (2017).

34 Deng, L. et al. First report of the human-pathogenic *Enterocytozoon bieneusi* from red-bellied tree squirrels (Callosciurus erythraeus) in Sichuan, China. *Plos One*. 11, (2016).

35 Liu, W. et al. First detection and genotyping of *Enterocytozoon bieneusi* in reindeers (Rangifer tarandus): a zoonotic potential of ITS genotypes. *Parasite Vector*. 8, 526 (2015).

36 Song, Y. et al. First report of *Giardia duodenalis* and *Enterocytozoon bieneusi* in forest musk deer (Moschus berezovskii) in China. *Parasite Vector*. 11, (2018).

37 Deng, L. et al. Molecular characterization and new genotypes of *Enterocytozoon bieneusi* in pet chipmunks (Eutamias asiaticus) in Sichuan province, China. *BMC Microbiol*. 18, (2018).

38 Yang, H. et al. Molecular characterization of *Enterocytozoon bieneusi* isolates in laboratory macaques in north China: zoonotic concerns. *Parasitol Res*. 116, 2877-2882 (2017).

39 Ye, J. et al. Occurrence of human-pathogenic *Enterocytozoon bieneusi*, Giardia duodenalis and Cryptosporidium genotypes in laboratory macaques in Guangxi, China. *Parasitol Int*. 63, 132-137

(2014).
40 Wang, S. et al. Seroprevalence of Toxoplasma gondii and *Encephalitozoon cuniculi* among domestic rabbits in central China. *Parasite*. 9, (2018).
41 Wang, X. T. et al. Multilocus genotyping of *Giardia duodenalis* and *Enterocytozoon bieneusi* in dairy and native beef (Qinchuan) calves in Shaanxi province, northwestern China. *Parasitol Res*. 115, 1355-1361 (2016).
42 Wu, J. et al. Prevalence, genotypes, and risk factors of *Enterocytozoon bieneusi* in Asiatic black bear (Ursus thibetanus) in Yunnan Province, Southwestern China. *Parasitol Res*. 117, 1139-1145 (2018).
43 Zhao, W. et al. *Enterocytozoon bieneusi* in sika deer (Cervus nippon) and red deer (Cervus elaphus): deer specificity and zoonotic potential of ITS genotypes. *Parasitol Res*. 113, 4243-4250 (2014).
44 Chen, W. et al. Natural infection rate of Microspore in bumblebees in four provinces of China. *Journal of Fujian Agriculture and Forestry University(Natural Science Edition)*. 295-300 (2010).
45 Li, J. et al. Occurrence, molecular characterization and predominant genotypes of *Enterocytozoon bieneusi* in dairy cattle in Henan and Ningxia, China. *Parasite Vector*. 9, 142 (2016).
46 Zhao, W. et al. Prevalence of *Enterocytozoon bieneusi* and genetic diversity of ITS genotypes in sheep and goats in China. *Infect Genet Evol*. 32, 265-270 (2015).
47 Zhao, W. et al. Genotyping of *Enterocytozoon bieneusi* (Microsporidia) isolated from various birds in China. *Infect Genet Evol*. 40, 151-154 (2016).
48 Tang, C. et al. Genetic diversity within dominant *Enterocytozoon bieneusi* genotypes in pre-weaned calves. *Parasite Vector*. 11, 170 (2018).
49 Deng, L. et al. Multi-locus genotypes of *Enterocytozoon bieneusi* in captive Asiatic black bears in southwestern China: High genetic diversity, broad host range, and zoonotic potential. *Plos One*. 12, (2017).
50 Ye, J. et al. Anthroponotic enteric parasites in monkeys in public park, China. *Emerg Infect Dis*. 18, 1640-1643 (2012).
51 Shi, K. et al. Molecular survey of *Enterocytozoon bieneusi* in sheep and goats in China. *Parasite Vector*. 9, (2016).
52 Karim, M. R. et al. Predomination and New Genotypes of *Enterocytozoon bieneusi* in Captive Nonhuman Primates in Zoos in China: High Genetic Diversity and Zoonotic Significance. *Plos One*. 10, (2015).
53 Qi, M. et al. Host specificity of *Enterocytozoon bieneusi* genotypes in Bactrian camels (Camelus bactrianus) in China. *Parasite Vector*. 11, (2018).
54 Zhao, W. et al. *Enterocytozoon bieneusi* in Dairy Cattle in the Northeast of China: Genetic Diversity of ITS Gene and Evaluation of Zoonotic Transmission Potential. *J Eukaryot Microbiol*. 62, 553-560 (2015).
55 Qi, M. et al. *Enterocytozoon bieneusi* Genotypes in Grazing Horses in China and their Zoonotic Transmission Potential. *J Eukaryot Microbiol*. 63, 591-597 (2016).
56 Li, W. et al. Human-Pathogenic *Enterocytozoon bieneusi* in Captive Giant Pandas (Ailuropoda melanoleuca) in China. *Sci Rep*. 8, 6590 (2018).
57 Huang, J. et al. New Genotypes of *Enterocytozoon bieneusi* Isolated from Sika Deer and Red Deer in China. *Front Microbiol*. 8, (2017).
58 Li, W. et al. High diversity of human-pathogenic *Enterocytozoon bieneusi* genotypes in swine in northeast China. *Parasitol Res*. 113, 1147-1153 (2014).

59 Li, W. et al. Presence of zoonotic *Cryptosporidium scrofarum*, *Giardia duodenalis* assemblage A and *Enterocytozoon bieneusi* genotypes in captive Eurasian wild boars (Sus scrofa) in China: potential for zoonotic transmission. *Parasite Vector*. 10, (2017).
60 Zhao, Y. et al. Investigation on Microspore infection in main crab raising areas of Jiangsu Province. *Journal of Aquaculture*. 11-14 (2017).
61 Ye, J. et al. Dominance of *Giardia duodenalis* assemblage A and *Enterocytozoon bieneusi* genotype BEB6 in sheep in Inner Mongolia,China. *Vet Parasitol*. 235-239 (2015).
62 Zhao, W. et al. High Prevalence of *Enterocytozoon bieneusi* in Asymptomatic Pigs and Assessment of Zoonotic Risk at the Genotype Level. *Appl Environ Microb*. 80, 3699-3707 (2014).
63 Li, W. et al. Genotypic distribution and phylogenetic characterization of *Enterocytozoon bieneusi* in diarrheic chickens and pigs in multiple cities, China: potential zoonotic transmission. *Plos One*. 9, e108279 (2014).
64 Ma, J. et al. Occurrence and molecular characterization of Cryptosporidium spp. and *Enterocytozoon bieneusi* in dairy cattle, beef cattle and water buffaloes in China. *Vet Parasitol*. 207, 220-227 (2015).
65 Hu, S. et al. Zoonotic and host-adapted genotypes of Cryptosporidium spp., *Giardia duodenalis* and *Enterocytozoon bieneusi* in dairy cattle in Hebei and Tianjin, China. *Vet Parasitol*. 248, 68-73 (2017).
66 Xu, H. et al. Genotypes of Cryptosporidium spp., *Enterocytozoon bieneusi* and *Giardia duodenalis* in dogs and cats in Shanghai, China. *Parasite Vector*. 9, (2016).
67 Liu, H. et al. Infection by and genotype characteristics of *Enterocytozoon bieneusi* in HIV/AIDS patients from Guangxi Zhuang autonomous region, China. *BMC Infect Dis*. 17, 684 (2017).
68 Zhang, W. et al. Genotyping of *Enterocytozoon bieneusi* and Subtyping of Blastocystis in Cancer Patients: Relationship to Diarrhea and Assessment of Zoonotic Transmission. *Front Microbiol*. 8, 1835 (2017).
69 Yang, J. et al. *Enterocytozoon bieneusi* genotypes in children in northeast China and assessment of risk of zoonotic transmission. *J Clin Microbiol*. 52, 4363-4367 (2014).
70 Wang, T. et al. First survey of Cryptosporidium, Giardia and Enterocytozoon in diarrhoeic children from Wuhan, China. *Infect Genet Evol*. 51, 127-131 (2017).
71 Wang, L. et al. Zoonotic Cryptosporidium species and *Enterocytozoon bieneusi* genotypes in HIV-positive patients on antiretroviral therapy. *J Clin Microbiol*. 51, 557-563 (2013).
72 Wang, L. et al. Concurrent Infections o*f Giardia duodenalis, Enterocytozoon bieneusi* , and *Clostridium difficile* in Children during a Cryptosporidiosis Outbreak in a Pediatric Hospital in China. *Plos Neglect Trop D*. 7, (2013).
73 Hu, Y., Feng, Y., Huang, C. & Xiao, L. Occurrence, source, and human infection potential of Cryptosporidium and *Enterocytozoon bieneusi* in drinking source water in Shanghai, China, during a pig carcass disposal incident. Environ Sci Technol. 48, 14219-14227 (2014).
74 Ma, J. et al. Human infective potential of Cryptosporidium spp., *Giardia duodenalis* and *Enterocytozoon bieneusi* in urban wastewater treatment plant effluents. *J Water Health*. 14, 411-423 (2016).
75 Huang, C. et al. Environmental Transport of Emerging Human-Pathogenic Cryptosporidium Species and Subtypes through Combined Sewer Overflow and Wastewater. *Appl Environ Microb*. 83, (2017).
76 Li, N. et al. Molecular Surveillance of Cryptosporidium spp., *Giardia duodenalis*, and *Enterocytozoon bieneusi* by Genotyping and Subtyping Parasites in Wastewater. *Plos Neglect Trop D*.

6, (2012).
77 Ye, J. et al. Zoonotic *Enterocytozoon bieneusi* in raw wastewater in Zhengzhou, China. *Folia Parasite*. 64, (2017).
